# A Comprehensive Account of Sound Sequence Imitation in the Songbird

**DOI:** 10.3389/fncom.2016.00071

**Published:** 2016-07-19

**Authors:** Maren Westkott, Klaus R. Pawelzik

**Affiliations:** Department of Theoretical Physics, University of BremenBremen, Germany

**Keywords:** mirror neurons, inverse problem, songbird, sensory motor learning, synaptic plasticity, precise spike times, recurrent networks, sequence learning

## Abstract

The amazing imitation capabilities of songbirds show that they can memorize sensory sequences and transform them into motor activities which in turn generate the original sound sequences. This suggests that the bird's brain can learn (1) to reliably reproduce spatio-temporal sensory representations and (2) to transform them into corresponding spatio-temporal motor activations by using an inverse mapping. Neither the synaptic mechanisms nor the network architecture enabling these two fundamental aspects of imitation learning are known. We propose an architecture of coupled neuronal modules that mimick areas in the song bird and show that a unique synaptic plasticity mechanism can serve to learn both, sensory sequences in a recurrent neuronal network, as well as an inverse model that transforms the sensory memories into the corresponding motor activations. The proposed membrane potential dependent learning rule together with the architecture that includes basic features of the bird's brain represents the first comprehensive account of bird imitation learning based on spiking neurons.

## 1. Introduction

Inverse sensor-motor models serve to generate a desired sensory input by appropriate motor actions. In this sense they attempt to “invert” the action-sensation mapping given by the physical world. While in general this mapping is not stationary, sound sequence imitation represents a comparatively well controlled situation. Therefore, it was tempting to propose inverse models as the mechanism enabling many bird species to imitate previously heard acoustic signals (Hanuschkin et al., 2013). The underlying hypothesis is that inverse models in the bird's brain perform a transformation of memorized sensory representations of sound sequences into spatio-temporal patterns of activities in motor areas that in turn generate the same sound sequences. This enables imitation of arbitrary sound sequences within the realm of the possible sounds the bird can produce. A crucial prediction of such so called causal inverse models are mirror neurons active during both singing as well as playback of a recording of the birds song. The responses of these mirror neurons to a playback would be delayed relative to the bird itself singing the song. This delay reflects the loop time it takes for motor activations to produce sound, which produces sensory activations that are looped back to the respective motor area (e.g., about 40 ms in zebra finches). Indeed, a recent study has found evidence for such delayed mirroring in area LMAN of the song bird (Giret et al., 2014) (for a review on auditory-vocal mirroring in songbirds see Mooney, 2014).

The unambiguously clear mirroring with roughly zero delay discovered in neurons in area HVC_*x*_ of swamp sparrows (Prather et al., 2008), however, is at odds with previous explanations using causal inverse models. It was suggested to reflect a “predictive inverse model” that could emerge from hebbian learning of a single stereotyped (i.e., predictable) song (Hanuschkin et al., 2013). If this was true, these neurons could not be (directly) involved in imitation of arbitrary sound sequences, i.e., their zero delay mirroring would represent a highly specific epiphenomenon emerging from a system enabling reproduction of a limited set of memorized sensory experiences of sounds.

Here, we propose an alternative causal inverse model in which zero-delay mirroring rather reflects a delayed feedback from motor areas backwards to HVC that compensates for the loop delay. The architecture consists of three interacting neuronal modules that could be identified with corresponding areas in the song bird involved in sound production. In particular, it includes the hypothetical feedback which for conceptual simplicity is realized by delay-lines (see **Figure 2A**).

The delayed feedback turns out to be particularly beneficial for solving the problem of learning inverse models when they are based on precise spatio-temporal spike patterns, because it can then be mapped to the problem of associative learning precisely timed spikes in response to spatio-temporal input patterns (chronotron learning, compare Gütig and Sompolinsky, 2006; Ponulak and Kasiński, 2010; Florian, 2012; Memmesheimer et al., 2014). We propose an extension on membrane potential dependent plasticity (MPDP), a recently proposed biologically plausible synaptic mechanism that was shown to be quite powerful for learning chronotrons (Albers et al., 2015). We show that our extended learning mechanism (MPDP^2^) not only solves the chronotron problem, but is similarly potent for learning the mappings of spatio-temporal spike patterns to spatio-temporal spike patterns as required in inverse models. In particular, we show that zero-delay mirroring in our model emerges in HVC_*x*_ neurons that by chance or because of anatomical constraints receive no direct memory input, but are rather activated via the feedback lines from the motor population *m* during singing.

For the reproduction of a song, a second problem needs to be solved: A memory template of spatio-temporal spike patterns needs to be learned from exposure to the tutor song. It is possible to imprint spatio-temporal spike patterns on recurrent networks such that, when the network is initialized with the beginning of a target sequence, the network's dynamics will associatively complete the full temporal sequence. There are plasticity rules for sequence imprinting (Memmesheimer et al., 2014), which, however, are quite artificial. Here, we show that the same mechanism that self-organizes the inverse model also allows for learning the synaptic connections in a recurrent network to robustly reproduce sequences of neuronal activations. We discuss the plausibility of the proposed integrative framework for explaining the sound sequence imitation capability and its experimentally testable predictions.

## 2. Materials and methods

### 2.1. Neuron model

In the first part of this study, we use two different neuron models to show that the learning algorithm we suggest here does not depend on the specifics of one particular neuron model to be capable of learning precise spike times in response to spatio-temporal input patterns. For simplicity, we use the simple integrate-and-fire model for all other parts.

#### 2.1.1. The simple integrate-and-fire neuron

We investigate plasticity processes in several different network setups. In each of these setups, a neuron *j* receives input from other neurons *i* via plastic synapses. We denote the time of the *k*-th spike of presynaptic neuron with index *i* as $t_{i}^{k}$. The neuron is modeled as a simple leaky integrate-and-fire neuron. We here use the formulation of the SRM_0_ model to facilitate the derivation of the plasticity rule (Gerstner and Kistler, 2002). The neuronal voltage *V*(*t*) is given by the sum of weighted synaptic input kernels ε(*s*) (postsynaptic potentials, PSPs) and reset kernels *R*(*s*), which model the neuronal reset after a spike. External input currents *I*_*ext*_(*t*) are low-pass filtered with a response kernel κ(*s*). The full equation reads

(1) $$\begin{array}{l} V(t)=\sum_{i}w_{i}\sum_{k}\varepsilon (t-t_{k}^{i}-t_{delay})+\sum_{t_{j}}R(t-t_{j})\\ \;+\int_{0}^{\infty }\kappa (t-s)I_{ext}(s)ds. \end{array}$$

Here, *w*_*i*_ is the weight from presynaptic neuron *j* to the postsynaptic neuron. κ = exp(−(*t*−*s*)∕τ_*m*_) is the passive response kernel by which external currents are filtered. We also include a delay of synaptic transmission *t*_*delay*_. The other kernels are

(2) $$\begin{array}{l} \varepsilon (s)=\Theta (s)\frac{1}{\tau_{m}-\tau_{s}}\left(\exp(-s/\tau_{m})-\exp(-s/\tau_{s})\right)\\ R(s)=\Theta (s)(V_{reset}-V_{thr})\exp(-s/\tau_{m}). \end{array}$$

τ_*m*_ is the membrane time constant of a LIF neuron determining the decay of voltage perturbations, and τ_*s*_ = 2*ms* is the decay time constant of synaptic currents, which turns up as the rise time of the PSP kernel. Θ(*s*) is the Heaviside step function. If there is no input, the voltage relaxes back to *V*_*eq*_ = 0. Spiking in this model is deterministic: If $V\left(t^{\prime }\right)=V_{thr}=20mV$, the neuron spikes and a reset kernel is added at time $t^{\prime }=t_{post}$. The formulation of the kernel makes sure that the voltage is always reset to *V*_*reset*_ = −60*mV* < *V*_*eq*_.

#### 2.1.2. The conductance based LIF neuron

The simple model above suffers from the fact MPDP^2^ (to be defined below) is agnostic to the type of synapse. In principle, MPDP Albers et al. (2015) can turn excitatory synapses into inhibitory ones by changing the sign of any weight *w*_*i*_. Since this is a violation of Dale's law, we present a more biologically realistic scenario involving MPDP^2^. We split the presynaptic population into *N*_*ex*_ excitatory and *N*_*in*_ inhibitory neurons. The postsynaptic neuron is modeled as a conductance based LIF neuron governed by

(3) $$\begin{array}{l} C_{m}\frac{\text{d}V}{\text{d}t}=-g_{L}(V-V_{L})-(g_{sl}+g_{f})(V-V_{h})-g_{ex}(V-V_{ex})\\ \;-g_{in}(V-Vin)\;, \end{array}$$

where *V* denotes the membrane potential, *C*_*m*_ = 0.16μ*F* the membrane capacitance, *V*_*L*_ = −70*mV* the resting potential, *g*_*L*_ = 20 the leak conductance, *V*_*i*_ = −75*mV* and *V*_*ex*_ = 0*mV* the reversal potential of inhibition and excitation, respectively and *g*_*in*_ and *g*_*ex*_ their respective conductances. The spike after-hyperpolarization is modeled to be biphasic consisting of a fast and a slow part, described by conductances *g*_*f*_ and *g*_*sl*_ that keep the membrane potential close to the hyperpolarization potential *V*_*h*_ = *V*_*i*_. When the membrane potential surpasses the spiking threshhold *V*_*thr*_ = −50*mV* at time *t*_*post*_, a spike is registered and the membrane potential is reset to *V*_*reset*_ = *V*_*h*_. All conductances are modeled as step and decay functions. The reset conductances are given by

(4) $$\tau_{f,sl}{\dot{g}}_{f,sl}=-g_{f,sl}+\Delta g_{f,sl}\sum_{t_{post}}\delta \left(t-t_{post}\right)\;,$$

where Δ*g*_*sl*_ = 5 resp. Δ*g*_*sl*_ = 1000 is the increase of the fast and slow conductance at the time of each postsynaptic spike. They decay back with time constants τ_*f*_ = τ_*s*_ < τ_*sl*_ = *C*_*m*_/*g*_*L*_. The input conductances *g*_*ex*_ and *g*_*in*_ are step and decay functions as well, that are increased by *w*_*i*_ when presynaptic neuron *i* spikes and decay with time constant τ_*s*_ = 2*ms*. *w*_*i*_ denotes the strength of synapse *i*.

### 2.2. The learning rule MPDP^2^

We derive the plasticity rule from the demand of a balanced membrane potential: the neuron should not be hyperpolarized nor too strongly depolarized. This is a sensible demand, because it holds the neuron at a sensitive working point and keeps metabolic costs down. To that end, we introduce two thresholds, ϑ_*P*_ < ϑ_*D*_ < *V*_*thr*_, between which we would like the membrane potential to stay. The weight change is chosen such that, whenever ϑ_*D*_ = 10*mV* is surpassed, all weights that contribute to the rise of the membrane potential are depressed, weighted by their respective influence given by the PSP-kernel ε. Whenever the membrane potential drops below ϑ_*P*_ = *V*_*L*_, all synapses that contribute to the membrane potential at that point in time are potentiated, such that for a repetition of the pattern the membrane potential is deflected to stay between bounds. Additionally, we bound the weights to stay below a maximum weight *w*_*max*_, symbolizing a maximal synaptic strength.

(5) $$\begin{array}{l} {\dot{w}}_{i}=\eta \left(w_{max}-|w_{i}|\right)\left(-\gamma {\left[V(t)-\vartheta_{D}\right]}_{+}+{\left[\vartheta_{P}-V(t)\right]}_{+}^{2}\right)\;\\ \;\sum_{k}\varepsilon \left(t-t_{i}^{k}-t_{delay}\right)\;. \end{array}$$

γ is a factor that scales inhibition to excitation. We choose γ = 650 for the simple integrate-and-fire neuron and γ = 150 for the concutance-based integrate-and-fire neuron.

Obviously, the PSP-kernel used in the learning rule only has a very direct interpretation in the case of simple integrate-and-fire neurons. However, in the learning rule, this term only serves to estimate the extent to which the postsynaptic membrane potential depends on the input of one particular neuron. Hence, we track and use the equivalent quantity as well for the case of the conductance-based Integrate-and-fire neuron (with τ_*m*_ = *C*_*m*_/*g*_*L*_). Furthermore, for the conductance-based Integrate-and-fire neuron, the upper threshold is chosen to lie between resting potential and firing threshold: ϑ_*D*_ = −53*mV*. For the inhibitory synapses coming into play for conductance-based neurons between the inhibitory presynaptic neurons and the output neurons, the learning rule is adapted such that the effect of learning on the membrane potential is preserved. To that end, we choose the same learning rule as for the excitatory synapses, just with the opposite sign:

(6) $${\dot{w}}^{inh}=-\dot{w}$$

#### 2.2.1. Chronotron

In this section, we introduce the basic setting for the Chronotron problem, a pattern classification task. In a simple feed-forward network, the output neuron is supposed to be trained to respond to sets of input patterns with spikes a particular moments in time and silence at all other times. To allow for this learning to be possible, a teacher signal has to be given to the output neuron to inform it, when to spike and when not to spike. In our model, this teacher signal is just a strong input. Consider a feed-forward network consisting of *N* = 200 presynaptic neurons and one postsynaptic neuron. For illustration purposes, each input neuron spikes once in each of the five patterns used during training. To train the output neuron to spike at a specific time in response to each input pattern, a single spike is induced at a fixed time *t*_*post*_ = 100*ms* by a supplementary external (teacher) input

(7) $$I_{ext}=a\exp\left(-\frac{t-t_{post}}{\tau_{s}}\right)\Theta (t-t_{post}).$$

*a* = 0.3 is the amplitude of the teacher input. The shape of the current is chosen to mimic a synaptic input and induce a PSP-like voltage perturbance (see Equation 2).

While in the case of the simple integrate-and-fire neuron, we consider synapses that can change signs, for the conductance-based positive and negative inputs need to be separated. To that end, the output neuron receives inhibitory input from *N*_*in*_ = 200 inhibitory presynaptic neurons and excitatory input from *N*_*ex*_ = 200 excitatory presynaptic neurons. An input pattern for learning thus consists of a set of one excitatory and one inhibitory input pattern. In each pattern, each input neuron spikes once.

#### 2.2.2. Inverse model

To investigate learning of inverse models, we construct a model of connected neuronal populations reflecting the brain anatomy of songbirds (see **Figure 2A**). A motor population *m*, which during learning is driven from an external source (not shown in the figure), activates the muscles in the syrinx for singing. The bird's cochlea converts sounds into activations sensory neurons in population *s*. We use a simple model of the sound generation and perception process (see below). *s* feeds into a sensorimotor area *sm* via plastic synapses that will be adapted to form the inverse model. A subpopulation *s*_2_*m*_2_ of *sm* sends a copy of its own activity into the motor area *m* via a monosynaptic connection. In the other subpopulation *s*_*x*_*m*_*x*_ of *sm*, also receiving sensory input from *s*_*sens*_ via synapses subject to the same learning rule, mirror neurons with zero delay form. In the songbird, *m* could be equivalent to RA, the source of noise equivalent to LMAN and *sm* could be a model of HVC, in particular could *s*_*x*_*m*_*x*_ be a model of HVC_*x*_ and the mirror neurons therein.

A population of *N*_*m*_ = 20 motor neurons in the motor area *m* via the model of the world feeds into *N*_*s*_ sensory neurons in population *s*. The membrane time constants of the neurons in the respective areas are $\tau_{m}^{m}=70ms$ and $\tau_{m}^{s}=8ms$. To model the bird hearing its own vocalizations, spatio-temporal activity in *m* is converted to input in *s* through several delayed linear transformations. We create *N*_*w*_ = 40 sparse matrices *M*_*r*_ with *r*∈1, …, 40, where each entry in a matrix is either zero, or a positve constant with probability *P*_*p*_ or a negative constant with probabitity $P_{n}=P_{p}=2.5\cdot 10^{-3}$. Spikes in the motor population at time *t* are denoted ${\vec{x}}^{m}$ with entries $\underset{k}{\sum }\delta \left(t-t_{i}^{k}\right)$, i.e., a sum of delta functions at spike times $t_{i}^{k}$ of neuron *i*. This is low-pass filtered by $\tau_{s}\overset{\cdot }{\vec{y}}=-\vec{y}+{\vec{x}}^{m}$.

The birds' auditory input into neuron *j* in the sensory population *s* from self-generated sounds is then given by a sum of linearly transformed versions of the motor activity with different delays differing by Δ_*delay*_ = 1*ms*

(8) $$I_{ext}^{j}(t)=\sum_{r\;=\;0}^{N_{w}}M_{r}^{j}\vec{y}(t-(r-N_{w}/2)\Delta_{delay}-\tau_{loop}).$$

This temporally spreads the self-generated sounds around the loop delay.

Mimicking the “babbling” young birds presumably use to establish the relation of motor activities with the corresponding sensations of self-generated sounds, the motor neurons are fed strong delta-shaped current pulses of height *h* = 0.5 with a frequency of *r*_*explor*_ = 1*Hz* (resp. *r*_*explor*_ = 2*Hz*) during an exploration phase. The firing rate of the neurons in motor population *m* is limited by the long hyperpolarization in this area introduced by the long membrane time constant $\tau_{m}^{m}$. This long hyperpolarization serves to suppress cyclic activity between areas *m* and *m*_2_*s*_2_.

Consequently, the spatio-temporal motor activity is transformed into input into the sensory neurons, which in turn create spatio-temporal sensory spike patterns.

Note that the sensory area *s* is split into two sub-populations *s*_*sens*_ and *s*_*recall*_ receiving the same input given by Equation (8). Only *s*_*recall*_ will be activated during recall, while *s*_*sens*_ only receives sensory input.

At all times, a copy of the motor activations in *m* is sent as teacher input to a population of sensorimotor neurons in *s*_2_*m*_2_ ($\tau_{m}^{sm}=8ms$) with a delay τ_*loop*_ equivalent to the loop delay. We simplify the possible complex process that provides a feedback to the sensorimotor population from the motor population to a simple delay line, considering only the resulting effective connection.

By this teacher input of strength *a* = 0.3, the spatio-temporal spike patterns activated in sensory memory area *s*_*recall*_ can be mapped back onto the delayed copy of the motor patterns in the sensorimotor population *s*_2_*m*_2_. The sensorimotor population *s*_2_*m*_2_ then gives a copy of its own activation as input into the motor population. The synaptic weights from the sensory population *s* to the sensorimotor population *s*_2_*m*_2_ are plastic according to Equation (5).

Before learning the inverse model, a target pattern is created by choosing one particular random pattern in motor area *m* as it would also be created during babbling with input rate *r*_*explor*_ and the respective sensory pattern, which are stored for later comparison to be able to evaluate the quality of the inverse model over time (for details see below). Over the course of learning, we evaluate the distance between the stored motor pattern and the motor pattern that is evoked when the tutor sensory pattern is fed into the sensory population (recall case). During recall, initially, only *s*_*mem*_ is activated by imposing the target pattern, which activates *s*_2_*m*_2_, which in turn activates *m* to—if the inverse model was successfully learned—reproduce the target motor pattern. We then use this motor pattern to test which sensory pattern it would evoke to be able to evaluate the relevant distance in sensory representations. Note that the activation in *m* entrails an activation of *sm* via the feedback loop and not generated from sensory input. This leads to the precise zero delay mirroring in *s*_*x*_*m*_*x*_.

To quantify the learning process, in each trial we measure the Victor- Purpura distance (Victor and Purpura, 1997) between tutor and recall spike patterns, while minimizing with respect to a global shift.

#### 2.2.3. Recurrent networks

Here, we investigate whether the same mechanism used for learning the inverse model can also serve to imprint spatio-temporal patterns on recurrent networks. For this purpose, we consider an all-to-all network of *N* neurons. During learning, all synapses within the network are considered to be plastic and to obey Equation (5). For proof of principle, the desired spatio-temporal patterns are taken to consist of *N* equidistant spikes with a distance of *d* = 2*ms*, where the order of spikes is randomly assigned. The patterns are looped twice during each learning epoch lasting *T* = 2*Nd* to allow for cyclic recall. All patterns are shown to the network in batch mode and then the synaptic weights are updated. The patterns to be memorized are fed into the network by giving the respective neurons an input which mimics a synaptic input from a different neuron population (see Equation 7) with height *a* = 0.3. During learning, all neurons receive additional gaussian noise of standard deviation σ = 0.5*mV*. Recall is performed without noise.

## 3. Results

In this study, we consider the same learning algorithm in three tasks: The chronotron problem, the learning of inverse models (see **Figure 2A**) and imprinting of spatio-temporal spike patterns onto recurrent networks.

### 3.1. The chronotron problem

We use the task of associative learning of precisely timed output spikes in response to specific spatio-temporal input patterns (the Chronotron task) to explain the fundamental learning mechanism that crucially depends on the dynamics of the membrane potential of the target neuron.

**Figure 1:**
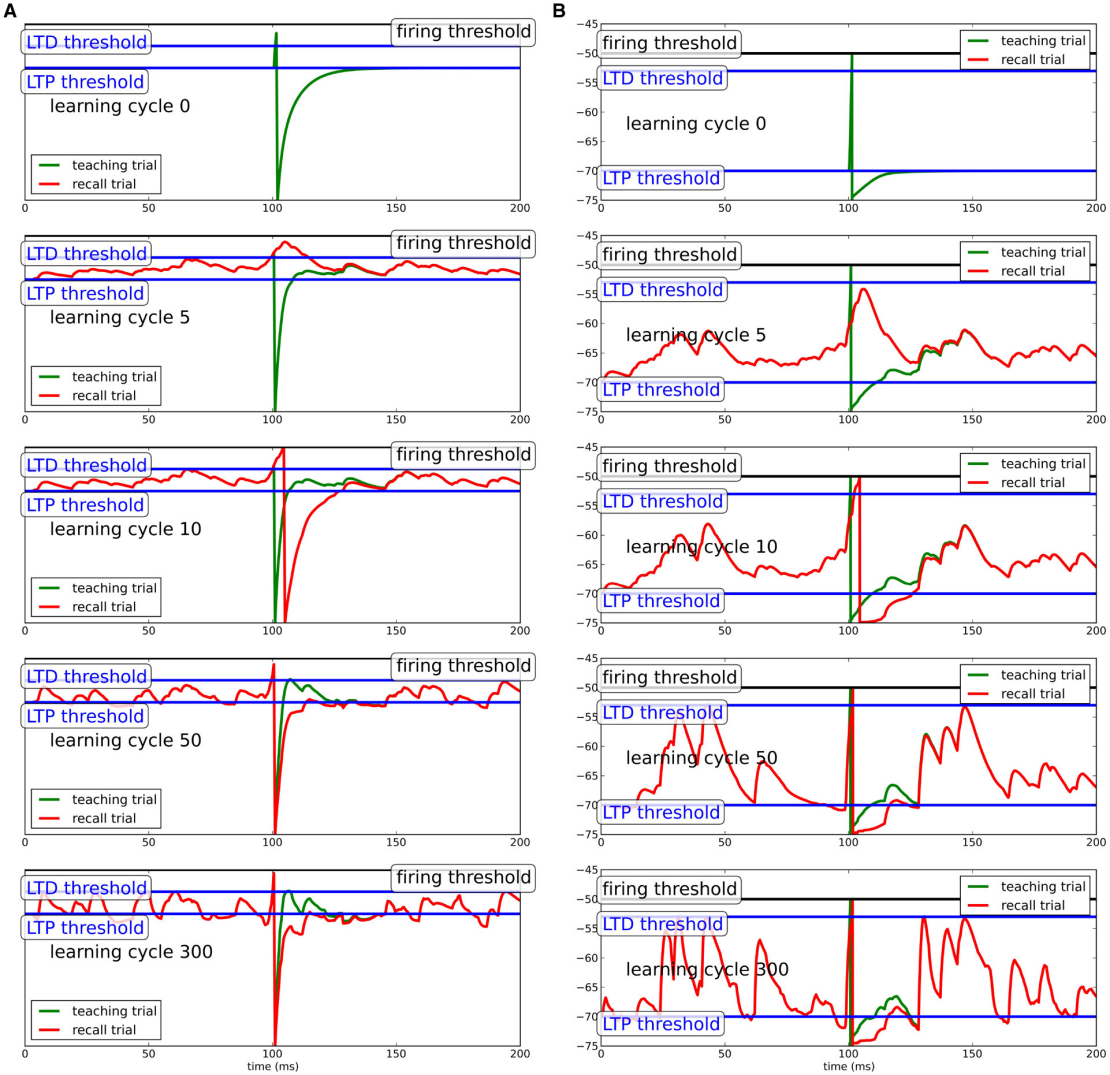
**Learning progress in Chronotron toy model for different neuron models. (A)** Simple integrate-and-fire neuron; **(B)** conductance-based integrate-and-fire neuron. During teaching (green line), a regular, but strong input evokes a spike at the desired spike time. Due to the hyperpolarization after the teacher spike, the neuron adapts its synapses to generate extra input around the spike, which produces a spike when the teacher input is omitted (recall trial, red line). Here, we show the learning progress for several different stages of learning. Precise spike times can be learned in response to several input patterns independent of the specifics of the model neuron.

To show that the learning mechanism is independent of the specifics of the neuron model, we show this for two different model neurons: In the conceptually simpler case, we use a simple Integrate-and-fire neuron (see Figure 1A) and in a more complex version, we use a conductance-based integrate-and-fire neuron (see Figure 1B).

The teacher input at *t*_*post*_ drives the neuron to spike. The membrane potential subsequently goes to the reset potential, from where it relaxes back to equilibrium. Since the reset potential is below the plasticity threshold ϑ_*P*_, synapses of presynaptic neurons that were active shortly before the output spike are potentiated to bend the membrane potential above the threshold for the next repetition of the input pattern. Likewise, when the membrane potential crosses the upper LTD-threshold ϑ_*D*_, synapses that were active shortly before will depress to bend the membrane potential away from the threshold. Note that for inhibitory synapses, potentiation and depression are reversed, such that the net effect of learning on the membrane potential is preserved. By this mechanism, the hyperpolarization after the spike will be filled up during learning (green trace in Figure 1). In the process, the membrane potential will become steeper just before the spike with synaptic input concentrated close to the teacher spike. This extra-input needed for compensation of the spike after-hyperpolarization during learning will, in absence of the teacher input, cause the neuron to spike shortly after the desired time. While the membrane potential becomes more and more deflected during learning, the output spike shifts a little bit forward in time and the hyperpolarization is more and more compensated. Since the potentiation of synapses depends on the distance of the membrane potential to the upper threshold quadratically and the depression of synapses depends on the distance of the membrane potential to the threshold linearly, for increasing additional input in the vicinity of the teacher spike, the contribution to potentiation shrinks more quickly than the contribution to depression, such that ultimately plasticity comes to a halt.

Note that for the simple integrate-and-fire neuron model, the hyperpolarization is very pronounced to provide a strong learning signal. For more sophisticated neuron models, the hyperpolarization does not need to be as pronounced, because it will not be linearly filled up during learning, such that the membrane potential can stay in a biologically realistic realm.

For the more sophisticated neuron models, inhibitory and excitatory synapses are separated. While this is necessary in biological systems, here, we collapse the input populations into a single one and allow for synapses to change sign in the simple integrate-and-fire neuron for simplicity. Since the learning rule only acts to influence the net effect of input on the membrane potential, the learning algorithm does not depend on the specifics of how the change in net effect is achieved.

Additionally, the simple integrate-and-fire neuron is advantageous for conceptual clarity and for speed of simulations. Hence, we will be using this model for the rest of this study.

### 3.2. Inverse model learning

**Figure 2:**
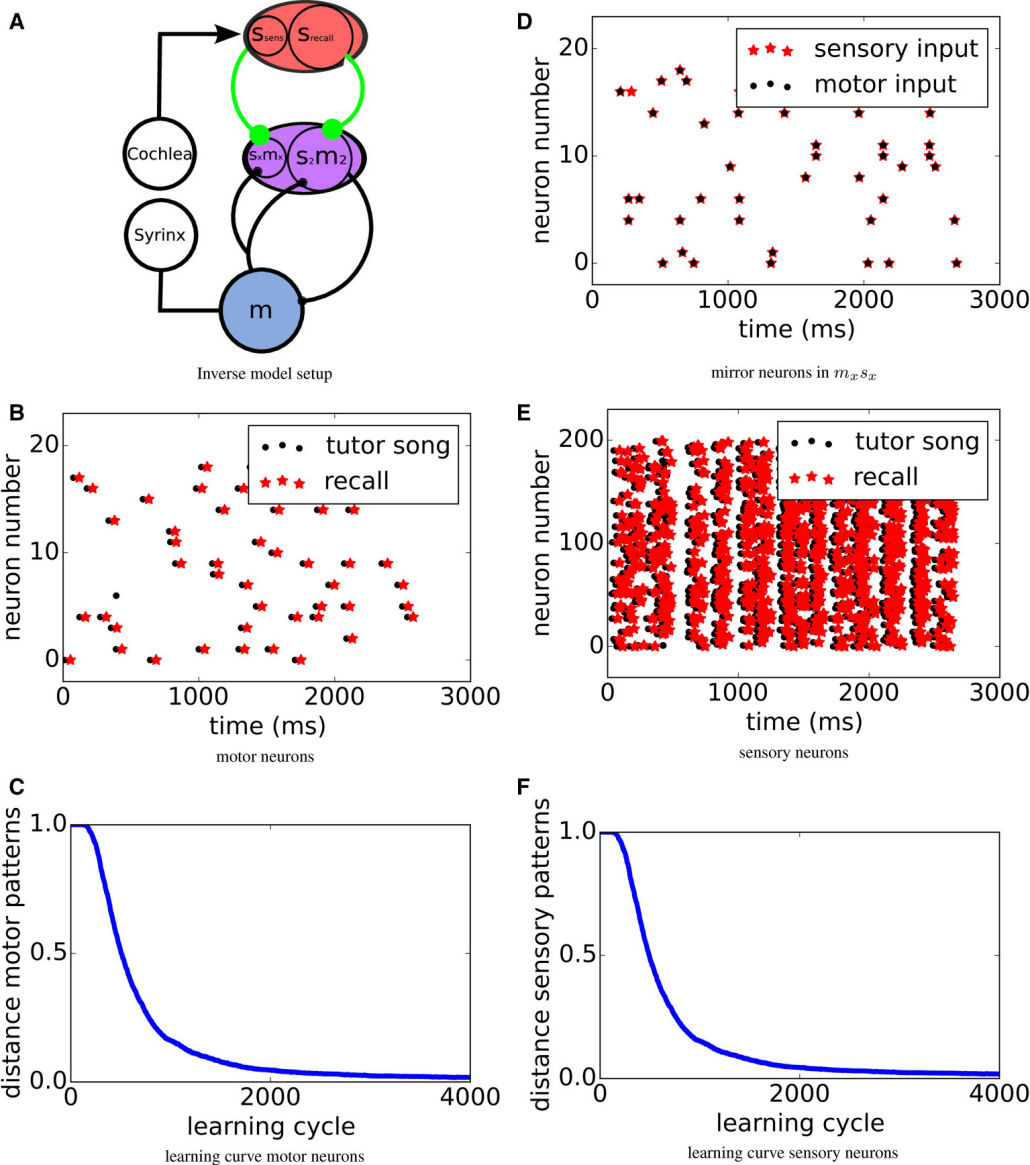
**(A)** Sketch of the system setup: A motor population *m*, which during learning is driven from an external source (not shown in the figure), activates the muscles in the syrinx for singing. The bird's cochlea converts sounds into activations sensory neurons in population *s*. *s* feeds into a sensorimotor area *sm* via plastic synapses (green) that will be adapted to form the inverse model by MPDP^2^. A subpopulation *s*_2_*m*_2_ of *sm* sends a copy of its own activity into the motor area *m* via a monosynaptic connection. In the other subpopulation *s*_*x*_*m*_*x*_ of *sm*, also receiving sensory input from *s*_*sens*_ via synapses subject to the same learning rule, mirror neurons with zero delay form. **(D)** Mirror neurons in *s*_*x*_*m*_*x*_. Black dots mark activity caused by motor input only, red stars activity caused by auditory input only. **(B,E)** Motor resp. sensory pattern. Black dots mark target spike times and red stars mark recall activity. For the sensory pattern, only half of the *N*_*s*_ = 400 sensory neurons are displayed. **(C,F)** Error in motor/sensory reproduction of target pattern over learning time. Learning curves for motor resp. sensory neurons. Learning converges quickly and robustly.

To investigate learning of inverse models, we construct a model of connected neuronal populations reflecting the brain anatomy of songbirds (see Figure 2A). A population *m* in the motor area activates the muscles in the syrinx for singing. The bird's cochlea converts sounds into activations of neurons in sensory area *s*. To model the bird hearing its own vocalizations, spatio-temporal activity in *m* is converted to input in *s* through several delayed linear transformations. This temporally spreads the self-generated sounds around the loop delay. Mimicking the “babbling” young birds presumably use to establish the relation of motor activities with the corresponding sensations of self-generated sounds, the motor neurons are driven with noise during an exploration phase (not shown in Figure 2A, see Figure 2B for an example pattern). Consequently, the spatio-temporal motor activity is transformed into input into the sensory neurons, which in turn create spatio-temporal sensory spike patterns (see Figure 2E).

A successful inverse model then has to map these sensory patterns, when retrieved from memory, back onto the relatively sparse motor patterns that have generated the respective sensory inputs, which is a task similar to the Chronotron task. Note that the sensory area *s* is split into two sub-populations *s*_*sens*_ and *s*_*recall*_ receiving the same input given by Equation (8). Only *s*_*recall*_ will be activated during recall, while *s*_*sens*_ only receives sensory input.

Before learning the inverse model, a target pattern is created by choosing one particular random pattern in motor area *m* and the respective sensory pattern, which are stored for later comparison to allow for comparison during the learning process. Since we choose the target pattern to be a particular pattern with the same statistics as the training set, it could by chance occur during the babbling phase. Due to the stochastic nature of the exploration, however, this is highly unlikely. This choice of target pattern is equivalent to assuming that the tutor bird has the same mapping from motor activity to sound and thus to auditory activity. We do this to ensure that the resulting song (i.e., sensory activation) can in principle be generated perfectly by our model bird.

During learning, we compare the stored motor pattern to the motor pattern that is evoked when the tutor sensory pattern is fed into the sensory population (recall case). We then use this motor pattern to test which sensory pattern it would evoke. Figures 2B,E show spike raster plots of the target motor resp. sensory activity (black dots) and recalled activity via the inverse model (red stars) for *r*_*target*_ = *r*_*explor*_ = 1*Hz*. After learning, the tutor pattern is very well reproduced in both the motor and the sensory area with a time delay of about τ_*loop*_. To quantify the learning process, in each trial we measure the Victor- Purpura distance (Victor and Purpura, 1997) between tutor and recall spike patterns, while optimizing for a global shift. The resulting error over the course of learning is displayed in Figures 2C,F. The error drops quickly and then settles on a low level.

**Figure 3:**
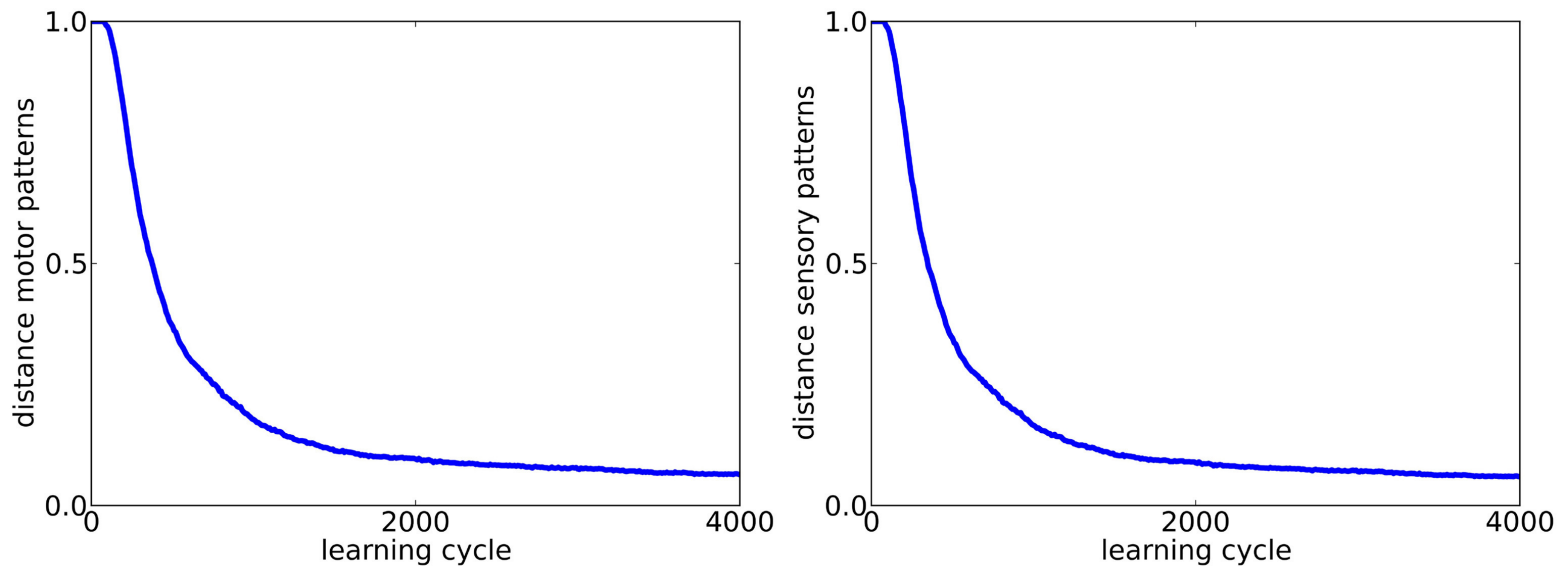
**Learning curves for motor (left) and sensory (right) population (***N***_***s***_ = 200) in the case of exploration with a firing rate (***r***_***explor***_ = 2***Hz***) different from the tutor song (***r***_***target***_ = 1***Hz***)**.

So far, exploration was done with the same firing rate as the tutor song, which raises the question if the forward mapping is in fact reversed or if the target pattern is just approximated during learning closely enough. To control for that, we investigate if tutor songs with a firing rate of *r*_*target*_ = 1*Hz* can also be reproduced if exploration is done with a firing rate twice as high *t*_*explor*_ = 2*Hz*. The respective learning curves are shown in Figure 3. We find that the target pattern is reproduced reasonably well and thus conclude that in fact, an inverse model is learned.

The above picture assumes that the neurons in *s*_2_*m*_2_ all receive their input from *s*_*recall*_. The bird's brain, however, contains also neurons in adjacent area *s*_*x*_*m*_*x*_ that receive their inputs from the primary sensory area *s*_*sens*_, as well as from the motor area *m*. We assume that here the same setting applies, i.e., that the feedback from *m* is delayed and the connections from *s*_*sens*_ to *s*_*x*_*m*_*x*_ are plastic according to the same learning mechanism. We find that then the neurons in *s*_*x*_*m*_*x*_ after learning indeed respond at similar times during both, active singing and passive listening, i.e., they represent zero delay mirror neurons as found experimentally in area HVC_*x*_ (Prather et al., 2008) (see Figure 2D). During singing its song, due to the activation in *m*, the same neurons in *s*_*x*_*m*_*x*_ are activated via the feedback lines at the same time relative to the song as they would be during passive hearing of the same song.

### 3.3. Recurrent network

Up to this point, we took for granted that an area feeding into or being identical with *s*_*recall*_ can reproduce sequences of spatio-temporal spike patterns representing the memories of previously encountered acoustic signals.

The extra-input into a neuron around each teacher spike from the other neurons in the network emerging during learning will make the membrane potential steeper around the spike and cause the spikes to shift forwards in time during learning. However, during learning the hyperpolarization, the signal for LTP, is slowly filled up, while at the same time the rise of the membrane potential before the spike becomes steeper. After the net effect of these two influences cancels, the learning comes to a halt.

Since after learning, around each spike for each neuron extra input is generated by the network, the network will fill in the missing teacher activity after learning, if the sequence is initialized by the first few spikes during an initialization period of *T*_*init*_ = 30*ms*. However, the resulting sequence may be stretched with respect to the original input sequence.

**Figure 4:**
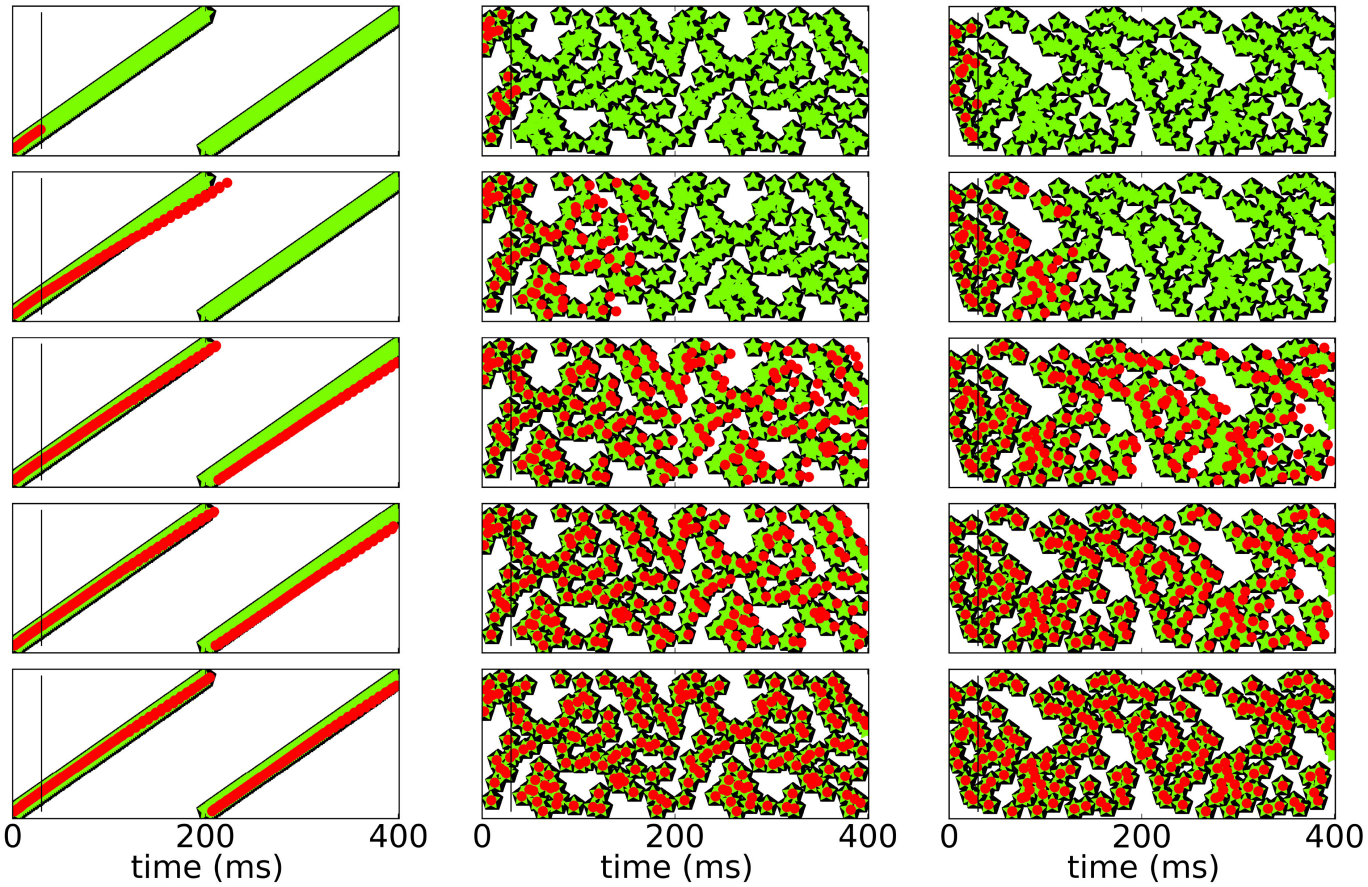
**Spike raster plots at different stages of learning**. Black pentagons are original activity of the network in response to the teacher input before learning, green stars are activity during learning and red dots are recall; the vertical black line demarks the end of the initialization sequence. From top to bottom after 100, 250, 800, 1200, 5000 learning cycles.

To test for stability of recall, we check if and for how long the network is able to reproduce the sequence and compare it to both, the initial response of the network to the input before learning and the response of the network after learning. Since spikes may shift over time, the sequence slightly changes. Figure 4 shows raster plots of three different patterns imprinted on the same neuronal network with *N* = 100 for *t* < *T* for different stages of learning.

**Figure 5:**
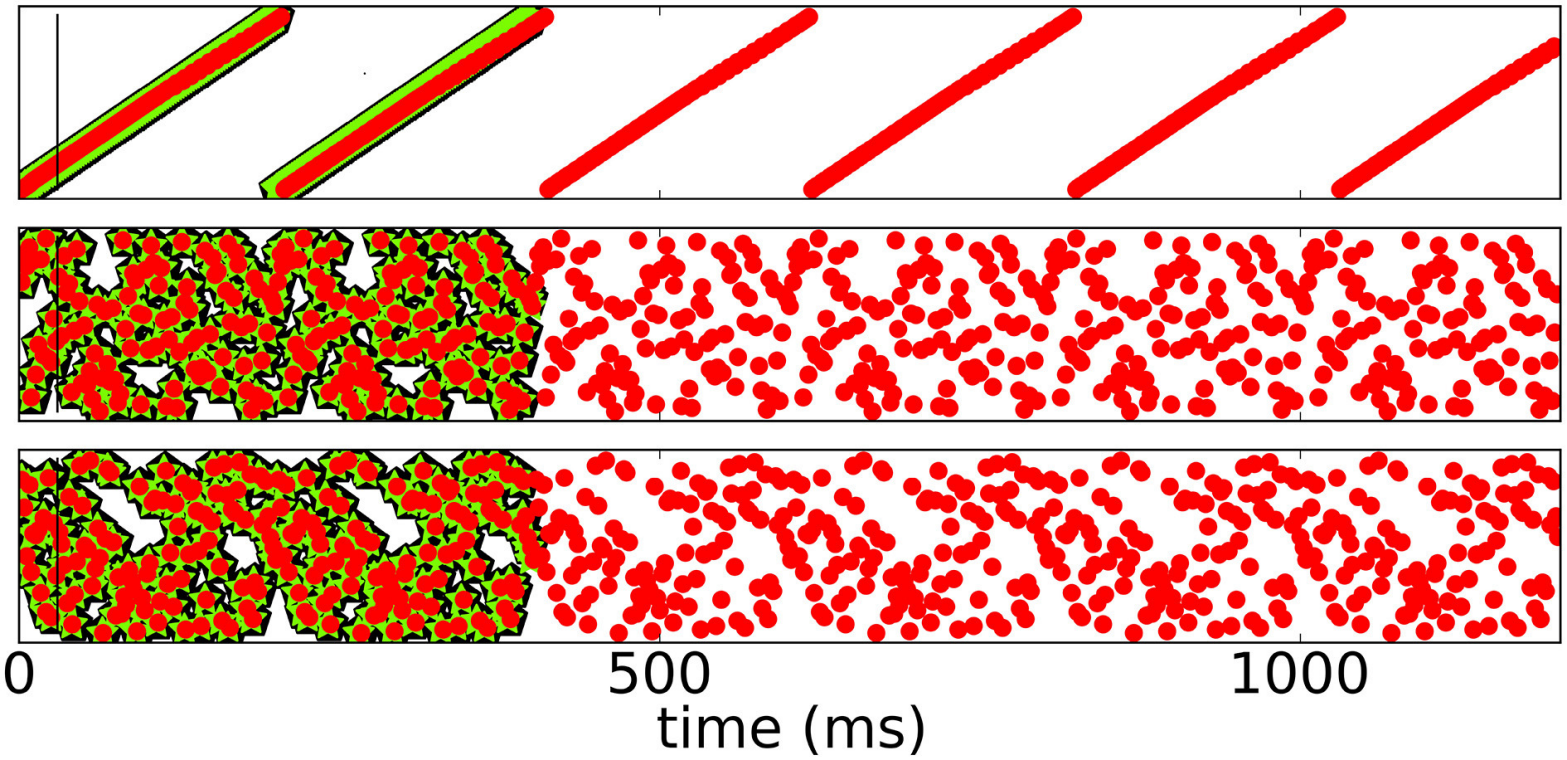
**Spike raster plots for long times after learning**. Black pentagons are original activity of the network in response to the teacher input before learning, green stars are activity during learning and red dots are recall; the vertical black line marks the end of the initialization sequence. The learned patterns are cyclic and stable after learning.

Figure 5 shows a rasterplot of the activity in the network during and after initialization for different patterns as it evolves for longer times. We tested recall over ten cycles, but here only show six for clarity. After learning, recall is cyclic and stable even for longer times.

We here only investigate the arguably simplest case of patterns that have only one spike per pattern. However, since we are able to imprint several of those patterns onto a network at the same time, we are confident that our learning mechanism is able to also learn more complex patterns.

## 4. Discussion

In this study, we introduce a homeostatic, biologically plausible learning rule for learning precise spike times in response to spatio -temporal spike patterns (Chronotron). We demonstrate this capability in a toy model feed-forward network. We then go on to show that our learning algorithm can also realize inverse models based on spatio-temporal spike patterns in a newly proposed network architecture based on the song system of the bird. Finally, we show that the same plasticity mechanism can serve to learn weights in recurrent networks to produce temporally precise sequences of spikes.

Synaptic plasticity is known to depend on the values of the membrane potential. The homeostatic objective of keeping the membrane potential between two bounds yields a learning rule (MPDP^2^) with a priori anti-hebbian characteristics. Paradoxically, the interaction with spike after- hyperpolarization turns this rule into a biologically plausible hebbian mechanism that allows for associative learning of precise spike times yielding a capacity for chronotrons that is comparable to more artificial rules (Albers et al., 2015). In particular, this mechanism can explain the hebbian spike timing dependent plasticity (STDP) of inhibitory synapses (Haas et al., 2006), as well as indications that also excitatory synapses can exhibit anti - hebbian STDP (Froemke et al., 2005; Sjöström and Häusser, 2006). We would like to point out that spike time learning with such a mechanism works irrespective of whether excitatory or inhibitory synapses are plastic, because only the net effect on the membrane potential matters for learning. We proposed a new variant of this mechanism (MPDP^2^) and showed that it can not only learn Chronotrons, but also connections in recurrent networks as well as inverse models. While we here do not investigate the storage capacity of the learning rule introduced here, we expect it to be in the same order of magnitude as MPDP (Albers et al., 2015).

Previous learning rules that accomplish learning of precise spike times in response to spatio-temporal spike patterns are supervised relying on a teacher signal which is provided from an outside entity. One class of these learning rules performs a perceptron classification on the static membrane potential (Xu et al., 2013; Memmesheimer et al., 2014). They are technical solutions that provide insight into the maximal capacity of Chronotron learning, but not into how learning could be implemented in real synapses. Another class of learning rules observes the entire output(s) of the neuron in response to the input spike pattern(s) and then evaluate them against the target output(s). Here, E- Learning (Florian, 2012) performs a gradient-decent on the Victor- Purpura distance (Victor and Purpura, 1997) between both spike trains. As a consequence, the weight changes associated with one spike can depend on distant output spikes. This strong non-locality in time is difficult to implement in a biological neuron. Another learning mechanism that was recently introduced is FP-Learning. Here, the output of the neuron is directly compared to the desired output, while allowing for a margin of error. When an output error is detected, the trial is interrupted and the weights contributing to that error are changed. Plasticity after the error is suppressed, which also would be difficult to implement in a real biological neuron.

All these learning rules suffer from no clear interpretation of how a comparison between the target state and the actual state of the neuron can realistically be achieved. As for the original MPDP (Albers et al., 2015), the modification explored here, MPDP^2^, provides such a mechanism in the combination of spike-after hyperpolarization with a homeostatic plasticity mechanism. The teacher provides information about the desired spike times by simply providing a strong input, thereby driving the output neuron to spike at the desired time. The subsequent after- hyperpolarization provides a means to compare the output generated by the network to the desired output without inducing spurious spikes detrimental for learning. The more the hyperpolarization is compensated by self-generated input, the closer the self- generated activity would be to the target output. In MPDP^2^, this emerges naturally: Potentiation is stronger for more hyperpolarized states, such that plasticity works to shift self-generated activity toward the target activity. On the other hand, subthreshold depolarization in absence of a teacher spike is suppressed which suppresses spurious spikes.

In the case of a simple integrate-and-fire neuron, the hyperpolarization needs to be very pronounced to provide a strong learning signal, which is at odds with biological realism. We show that in a more realistic neuron model, such as a conductance-based integrate-and-fire neuron, this overly strong hyperpolarization is not necessary, because a sufficiently strong hyperpolarization that becomes filled non-linearly during learning can be provided by an additional conductance. We found that the general learning principle introduced here allows for learning of precise spikes also in Hodgkin-Huxley-type neurons, if the influence of the additional learning signal provided by the spike is clipped (not shown).

Suppression of spurious spikes is particularly important in learning of recurrent networks, since there, additional spikes would cause the entire pattern to fail. Here, we show that MPDP^2^ is also suitable for learning of sequences in recurrent networks.

The introduction of noise during learning has been shown before to be beneficial for stability by Laje and Buonomano (2013). In their study, Laje and Buonomano showed that in a network of rate neurons, stable activation patterns can be learned, if innate activation patterns of the network were additionally trained with the addition of noise. This generates an attractor around the desired activation pattern sequence. Using innate patterns that the network would generate spontaneously before learning has the added benefit of guaranteeing that the pattern can in fact be learned. In our study, the slight variability of the spiking pattern due to the shifting of individual spikes during the learning process, which is a result of a real teacher input, may be similarly beneficial for the selection of patterns that can be learned. In the model presented here, additive noise is used as well for the stabilization of the recall yielding stable sequences similar to what was shown in studies in non-spiking neurons (Jaeger and Haas, 2004; Sussillo and Abbott, 2009).

We applied an algorithm that was originally devised for Chronotron learning to learning in recurrent networks. This is based on the insight that for each individual neuron, the situation in both setups is very similar: The neuron has to produce precisely timed spikes in response to a given input pattern, which in the case of recurrent networks is generated by the rest of the network. Another algorithm devised for Chronotron learning (FP-learning) has been successfully applied to the learning in recurrent networks before Memmesheimer et al. (2014). There, the finite precision that is required during the learning process allows the spikes to slightly move around during the learning process, which may be similarly effective for stabilizing sequences as the addition of noise.

The interplay between the neuronal dynamics in the form of the hyperpolarization and the learning rule is particularly important for the learning mechanism. In their study, Brea et al. (2013) similarly propose that the learning algorithm should match the neuronal dynamics. They devise a learning rule starting from the given dynamics of the neuron, which optimizes the recall properties after learning. However, their learning algorithm only serves to approximate a given target spiking distribution, and thus does not operate on single spikes.

Both, the learning rule proposed by Brea et al. (2013) and FP-Learning Memmesheimer et al. (2014) suffer from an unclear interpretation of the teacher input. Here, in contrast, the teacher input is just a regular synaptic input, albeit of high amplitude.

These favorable properties of MPDP_2_ are also shared by the original MPDP (Albers et al., 2015). However, MPDP was conceived for a rather abstract teacher signal of arbitrary strength, which is nice for pattern classification, but does not provide enough flexibility of spiking times during learning for learning in recurrent settings. MPDP_2_, however, allows for spikes to slightly shift forward in time during learning, allowing for stability also in the absence of the (extra) teacher input.

We then go on to show that our learning algorithm can also realize inverse models based on spatio-temporal spike patterns. We use this in a new architecture consisting of coupled neuronal networks mimicking the anatomy of the songbird's brain to explain the reproduction of previously encountered acoustic signals. Crucially, it assumes the existence of delayed feedback from a motor area to a sensorimotor region which serves as an intermediate relay for realizing the inverse model that has been suggested to underlie the bird's sound imitation capabilities. In our model, we assume a simple delay line to provide this feedback. Of course, in the songbird, the feedback would be realized in a more complex way, which we here simplify to only consider the effective connection. The existence of zero delay mirror neurons in birds (Prather et al., 2008) provides strong evidence for such a feedback. Note, that in our model the zero delay mirror neurons represent an epiphenomenon which serve no function. In fact, in our model zero delay mirror neurons naturally emerge from the same learning mechanism acting on synapses that feed only sensory input, but not memory input, into areas that also receive delayed feedback. Interestingly, the singing related activity of the zero-delay mirror neurons found in HVC is not distorted by acoustic manipulations disrupting auditory feedback during singing, suggesting that these neurons receive purely motor- related input when the bird sings (Prather et al., 2008).

Furthermore, our model predicts mirror neurons with a delay equivalent to the loop delay in the respective sensory and motor areas involved in recall. This delay was linked to causal inverse models before (Hanuschkin et al., 2013). In fact, experimental evidence for this type of delay was found, albeit in songbird brain area LMAN (Giret et al., 2014). In their study, Hanuschkin et al. (2013) suggest a simple hebbian learning rule, which relies on the comparison between self-generated and target input. As for the learning rules for Chronotron learning discussed above, there is no clear biological interpretation of how this comparison could be done. Additionally, in their study, they only discuss a linear model of sound generation and perception which is local in time suitable for analytical tractability. In the songbird, however, the process of sound generation and perception can be assumed to be both, non-linear, and non-local in time. In the present study, we include non- linear spiking neurons for sound perception as well as for the motor-sensory mapping that includes interactions that are not local in time.

Our model requires a switch to suppress singing activity during passive playback, which could be implemented by strong inhibitory input into the motor area *m*. This hypothesis is supported by experimental evidence, since while in awake birds, motor neurons downstream of HVC are not responsive to auditory stimuli, in anesthetized birds, playback of the birds own song excites neurons in all nuclei in the song system downstream of HVC (Doupe and Konishi, 1991; Dave and Margoliash, 2000; Sturdy et al., 2003).

In contrast to other models (Hanuschkin et al., 2013), however, we do not need auditory input to be explicitly gated off during singing, because echoing is automatically suppressed by the self-inhibition induced by long hyperpolarizations in the motor area *m*. This self-inhibition comes with a characteristic dip of the length of the loop delay in the spike auto-correlation of the involved motor neurons, which could be experimentally accessible.

In conclusion, our results suggest that MPDP^2^ provides a unique and novel learning mechanism for establishing both, inverse models as well the connections in recurrent networks that putatively underlie the memories of sound sequences. Together with the novel architecture, the proposed membrane potential dependent learning mechanism to our knowledge provides the first comprehensive account of sound sequence imitation in birds that is completely based on sub-millisecond precise spatio-temporal spike patterns.

## Author contributions

This study was conceived and designed in collaboration between MW and KP. The numerical work was done by MW. Interpretation and discussion of the data was a joint effort by MW and KP, as was the writing process.

## Funding

MW was funded by the German Ministry of Science and Education (BMBF), grant number 01GQ0964.

### Conflict of interest statement

The authors declare that the research was conducted in the absence of any commercial or financial relationships that could be construed as a potential conflict of interest.
